# Investigating microbiome and transcriptome data to uncover the key microbial community involved in lignocellulose degradation within the Deulajhari hot spring consortium

**DOI:** 10.1016/j.dib.2023.109648

**Published:** 2023-10-05

**Authors:** Sangita Dixit, Kalpana Sahoo, Mahendra Gaur, Enketeswara Subudhi

**Affiliations:** a Centre for Biotechnology, School of Pharmaceutical Sciences, Siksha ‘O’ Anusandhan (Deemed to be University), Bhubaneswar, India; b Drug Development and Analysis Laboratory, School of Pharmaceutical Sciences, Siksha ‘O’ Anusandhan (Deemed to be University), Bhubaneswar, India

**Keywords:** Hotspring, Consortium, Microbiome, Metagenomics, Metatranscriptomics, Lignocellulose

## Abstract

Geothermally heated spring water contaminated with decomposed leaf biomass creates unique hot spring ecosystems that support the recycling of diverse nutrients and harbor microbial consortia capable of degrading lignocellulose. We present microbiome and transcriptome data from the bacterial consortium of Deulajhari hot springs, characterized by a temperature of approximately 58 °C and surrounded by a dense population of pandanus plants in Angul, Odisha, India. Metagenomics and metatranscriptomics datasets were generated by extracting total DNA and RNA from the consortium sample of hotspring sediment, followed by shotgun sequencing using the Illumina HiSeq 2500 platform. The metagenomics dataset produced approximately 38,694 contigs, while the metatranscriptomics dataset yielded 9226 contigs, resulting in a total nucleotide size of 89,857,616 and 15,541,403 bps, respectively. Analysis using MEGAN6 against the NCBI “taxonomy” database revealed the presence of 18 and 12 phyla, including candidate phyla, in respective datasets. *Proteobacteria* exhibited the highest relative abundance in the metagenomics dataset, while *Firmicutes* was highly abundant in the metatranscriptomics dataset. At the genus level, a total of 92 and 25 genera were predicted in both datasets, with lignocellulose degrading *Meiothermus* being highly abundant in both metagenomics and metatranscriptomics datasets. We also observed that the unknown bacteria and unidentified sequences were found in significant proportion in the metatranscriptomics dataset. We assembled and functionally annotated approximately 23,960 contigs using the Prokka pipeline. Among the SEED category, the most expressed and annotated microbial genes fall under the unknown category as well as Biotin synthesis and their utilization. Furthermore, some of these genes were implicated in the degradation of aromatic amino acids, D-mannitol, and D-mannose. These findings contribute to our understanding of how the composition and abundance of bacterial communities facilitate lignocellulose degradation in extreme environments and biofuel generation.

Specifications Table

**Table:** 

Subject	Microbial Ecology, Genomics and Molecular Biology
Specific subject area	The consortium samples prepared from sediment of the extreme environment condition contaminated with leaf biomass were analyzed using metagenomics and metatranscriptomics
Type of data	WGS, RNA-Seq, Table, Figure
How the data were acquired	Three replicates of sediments, weighing 200-300 grams were collected from the Deulajhari hot spring cluster. The consortium was prepared using pandanus leaf biomass as the carbon source for bacteria. A 4 ml sludge slurry was obtained from the thermophilic consortium sample for the extraction of DNA and RNA simultaneously. For sequencing, an Illumina HiSeq 2500 instrument was utilized to acquire shotgun metagenomic and metatranscriptomic sequence data. The raw data was underwent pre-processing, including the removal of low-quality data, before being subjected to primary sequence analysis. The generated primary data was subjected to taxonomic and functional analysis using the MEGAN6 software.
Data format	Raw: FASTQ files Analyzed: Graph, Tables and figures of the annotated data
Description of data collection	Sediments (200-300 gm from ∼4-5 feet depth) in 3 replicates were collected to prepare consortium by using pandanus leaf (0.5% w/v), 100 ml BHB media. The mixture was subjected to continuous shaking at 200rpm at 55 °C for 72 hours. This process was repeated for three batches/weeks to acclimatise the consortium. 10 ml aliquot was transferred from one batch to next. 4 ml sludge slurry was extracted for DNA and RNA extraction followed by sequencing using the Illimina HiSeq 2500.
Data source location	Sediment samples were collected from the Deulajhari hotspring (20°44’38.997” N, 84°29’49.8624” E, January 2021) situated in Anugul district in Odisha, India. A bacterial consortium sample was prepared at the laboratory of the Centre for Biotechnology, Siksha O Anusandhan (Deemed to be University).
Data accessibility	Data are given in this article. Raw metagenomic and metatranscriptomic data have been deposited in the Sequence Read Archive of the National Center for Biotechnology Information NCBI with the accession id: SRR23269503 (Metagenomics) and SRR23269502 (Metatranscriptomics). Direct link: https://www.ncbi.nlm.nih.gov/sra/SRR23269503 https://www.ncbi.nlm.nih.gov/sra/?term=SRR23269502

## 1 Value of the Data

• First attempt to employ metagenomics and metatranscriptomics to study the genetic diversity of hot spring consortium communities and their expression analysis.
• The generated sequence data can be used for performing comparative taxonomic and functional analyses alongside other metagenomes available in the database.
• The secondary analysis of the sequence data may provide information regarding polysaccharides degrading genes
• The leads may employed for the prospect of exploring suitable agri-waste biomass degrading enzymes for generating biofuels through cultivable methods.

## 2 Objective

In this study, pandanus leaf biomass served as the exclusive carbon source for lignocellulose degradation utilizing a microbial consortium extracted from the sediment of the Deulajhari hot spring. Metagenomic and metatranscriptomic profiling techniques were employed to gain insights into the composition and abundance of bacteria, genes as well as active enzymes involved in lignocellulose degradation. This investigation holds significant importance as it sheds light on the potential of the Carbohydrate-Active enZymes (CAZymes) identified within our hot spring consortium for biofuel production

## 3 Data Description

**Fig. 1:**
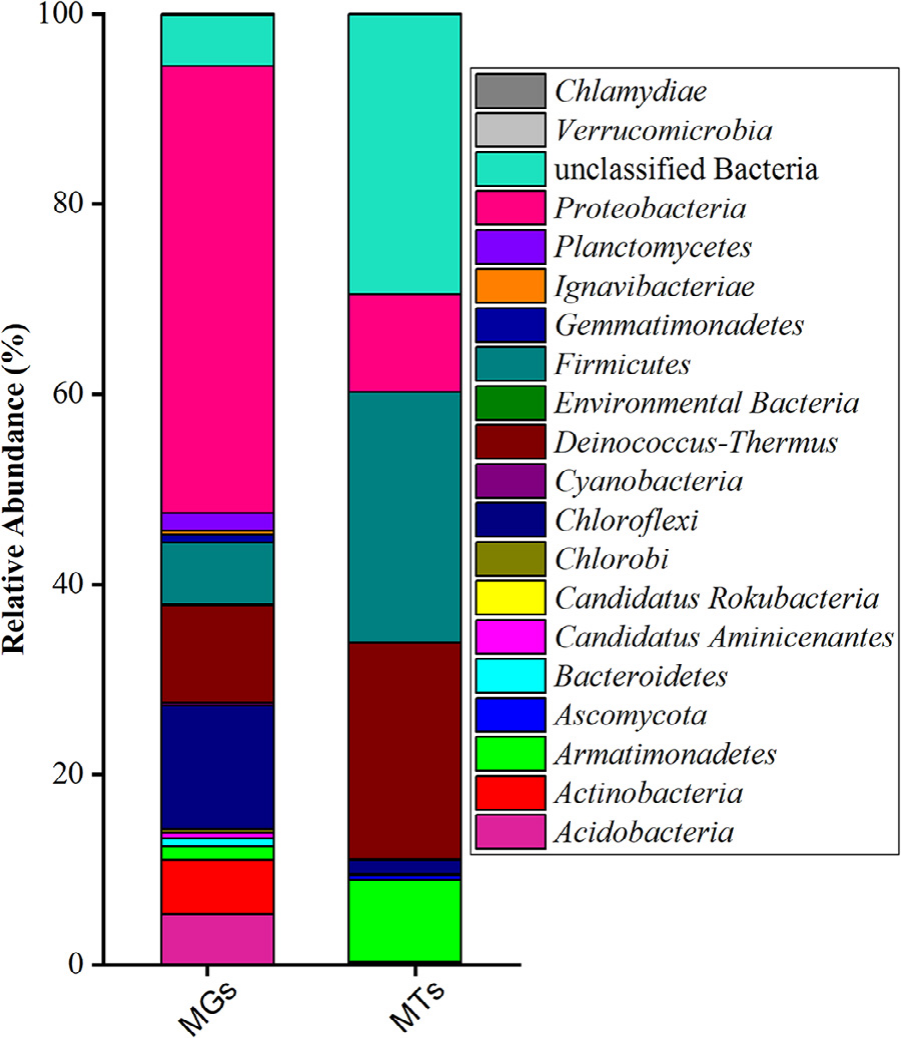
Stacked bar plots showing the relative abundance and overall transcriptional activities of dominant phyla in metagenomes (MGs) and metatranscriptomes (MTs) from the thermophilic cellulose-degrading microbial communities.

**Fig. 2:**
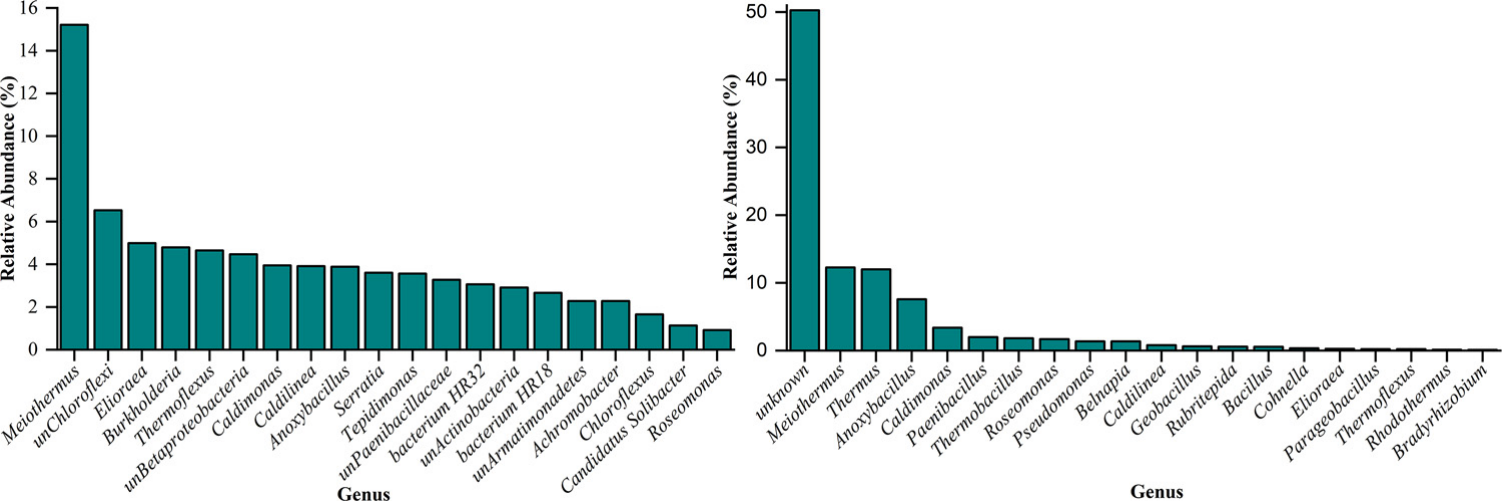
Relative abundance and overall transcriptional activities distribution of dominating order in the metagenomic (left) and metatranscriptomic (right) profile of all the datasets at genera levle.

**Fig. 3:**
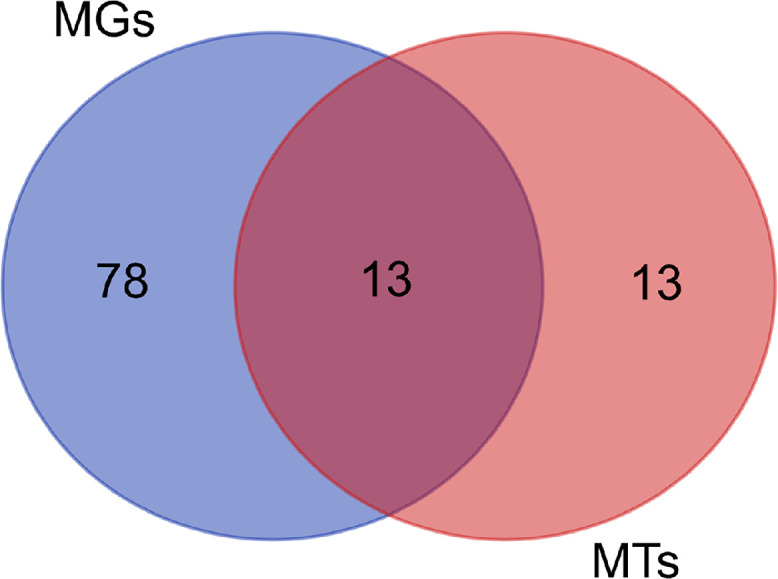
Venn diagram showing the number of unique and shared OTUs for the metagenomics (MGs) and metatranscriptomics (MTs) datasets.

A total of 2.27 gb of metagenomic and 7.19 gb of metatranscriptomic raw reads were generated from Illumina sequencing. After quality control, 15,297,032 paired-end clean data from the metagenome and 86,977,624 from the metatranscriptome were obtained. The GC% for the metagenome was 64.34%, while for the metatranscriptome, it was 57.66% (Table 1). Bacteria were identified as the dominant group, accounting for 80.58% of the metagenomic dataset, while viruses contributed a lesser portion (19.41%) whereas 28.7% and 69.08% accounting for unknown sequences and bacteria in metatranscriptomics datasets respectively (Table S1). Thermophilic bacteria *Firmicutes, Deinococcus-Thermus, Proteobacteria* and *Armatimonadetes* were detected at the phylum level (Fig. 1) and at genus level *Meiothermus, Paenibacillus, Roseomonas, Belnapia, Caldilinea, Rubritepida, Thermus* and *Brevibacillus* were observed in metagenomics and metatranscriptomics datasets (Fig. 2). The phyla *Proteobacteria* observed the highest abundance (46.96%) in the metagenomics dataset whereas *Firmicutes* was found to be the highest abundant (26.37%) in metatranscriptomics datasets (Fig. 1). Approximately 13 OTUs at the genus level were found to be common among the two datasets, whereas 78 and 13 genera were uniquely present in metagenomics and metatranscriptomics datasets respectively as shown in Fig. 3. However, 5.38% and 29.43% of the identified bacteria in both datasets remained unclassified (Table S2).

**Table 1:** Statistics table for Denovo assembly and annotation for Metagenome Metatranscriptome dataset.

Features	Metagenome	Metatranscriptome
Contigs (≥0 bp)	93140	51866
Contigs (≥1000 bp)	13263	4372
Contigs (≥5000 bp)	2109	285
Contigs (≥10000 bp)	1123	93
Contigs (≥25000 bp)	464	41
Contigs (≥50000 bp)	206	17
Total length (≥0 bp)	110353573	23557505
Total length (≥1000 bp)	72577435	12138669
Total length (≥5000 bp)	52392262	4420201
Total length (≥10000 bp)	45528837	3150486
Total length (≥25000 bp)	35354291	2362173
Total length (≥50000 bp)	26442403	1467298
Contigs	38694	9226
Largest contig	1297203	148234
Total length	89857616	15541403
GC (%)	64.34	57.66
N50	10600	2201
L50	1066	1358
Number of genes	88642	16276
Number of rRNAs	33	18
Number of tRNAs	1151	244
Number of tmRNAs	21	3

**Fig. 4:**
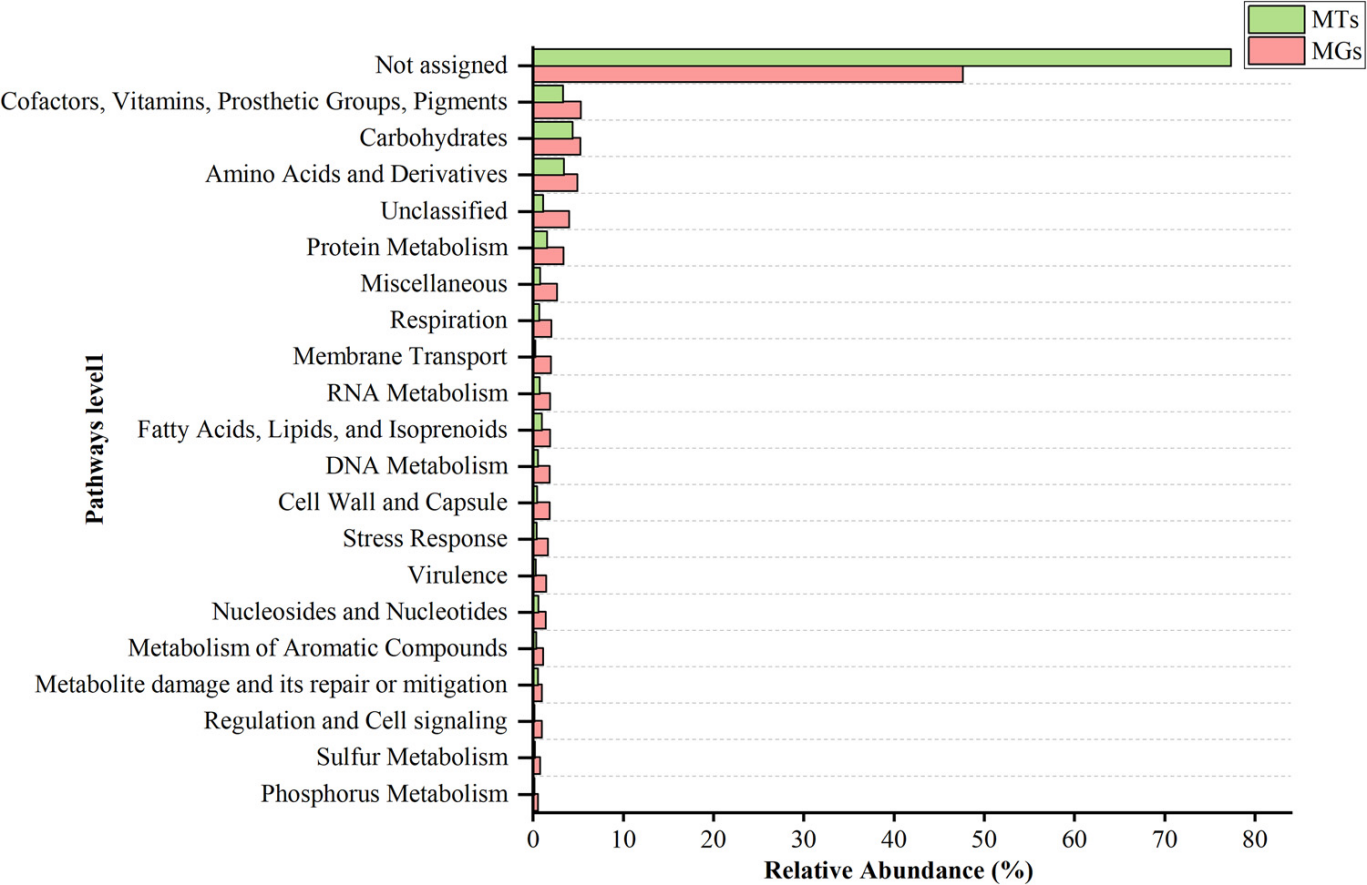
Functional SEED analysis using Fisher's exact test and 0.95 confidence interval of metagenome (MGs) and metatranscriptome (MTs) dataset depicting the major pathways enriched in the consortium sample of Deulujhari hotspring.

Using Prokka-annotated open reading frames [1], we predicted approximately 88,642 coding sequences (CDS) in the metagenomics dataset and 16,276 CDS in the metatranscriptomics dataset. Furthermore, the metagenomics and metatranscriptomics datasets exhibited 33 and 18 predicted ribosomal RNAs (rRNA), along with 1151 and 244 predicted transfer RNAs (tRNA), respectively. The total number of gene families identified in both datasets was 88,642 and 16,276, respectively. Functional annotation of all the contigs was carried out by SEED classification (Table 1). From the functional annotation, 38,694 contigs were assigned to 40 pathways (level-1), 673 super pathways (level-2), and 686 sub-pathways (level-3) (Table S3). Like that in metatranscriptomics datasets, 51,866 contigs were assigned to 43 pathways (level-1), 717 super pathways (level-2), and 1149 sub-pathways (level-3) were found through SEED classification (Table S3). Notably, the highest gene representation was observed in Amino Acids and Derivatives (9339 and 1869), Carbohydrates (7454 and 1869), Cofactors, Vitamins, Prosthetic Groups, Pigments (6739 and 1817), and Protein Metabolism (4408 and 840) in the metagenomics and metatranscriptomics datasets (Fig. 4). Moreover, some of these genes were implicated in Aromatic amino acid degradation and D-mannitol and D-mannose degradation in plants (Table S3). The SEED classification of metagenomics and metatranscriptomics datasets is illustrated in Fig. 4**.** The number of reads assigned to each function is given in Supplementary Table S3.

## 4 Experimental Design, Materials and Methods

### 4.1 Sediment and leaves sampling

For sample collection, a specialized stainless-steel scraper was employed. The leaf of the pandanus tree (*Pandanus odoratissimus*), which was near the springs and functioned as a carbon source, was collected as well. Both the sediment and leaf samples were immediately collected, sealed in sterile zipper polythene bags, and transported within 24 hours (hrs) to the laboratory under refrigeration. A Checktemp^Ⓡ^ water-resistant digital thermometer (Hanna Instrument, USA), and portable pH meter (Hanna Instrument, USA) were used to monitor the hot spring's temperature and pH on-site, respectively.

### 4.2 Development of consortium through pandanus leaf litter

There were several preparatory processes used for the pandanus leaf biomass. To remove any remaining moisture, it was first washed with ddH_2_O and then dried for 72 hrs at 50 °C. The dried biomass was divided into pieces around 4 × 4 mm in size to increase the surface area available for interaction with lignocellulose enzymes generated by the bacterial community. Then, a sterilized 500 ml flask containing 100 ml of BHB (HiMedia, USA) media and a sediment sample were filled with the leaf pieces (0.5% w/v). For 72 hrs, the flask was continuously shaken at 200 rpm at 55 °C using a rotary shaker (New Brunswick Scientific, USA). To allow the consortium time to adjust, this procedure was done in three subsequent batches at intervals of 4–5 days each. An aliquot from the previous batch was transferred in 10 ml throughout each cycle. The final batch's resultant supernatant, also known as the crude cellulose enzyme, was collected for an enzyme assay following a 72-hour incubation period. The 3,5-dinitro salicylic acid (DNSA) method, which is detailed in the article [2], was used to carry out the enzyme assay. In a nutshell, 1 ml of the supernatant was combined with 0.5 ml of DNS reagent and prepared for 15 minutes at 90 °C in a water bath. A UV-visible spectrophotometer (Thermo Scientific, USA) was then used to estimate the amount of emitted reducing sugar at 540 nm.

### 4.3 Total DNA and RNA extraction

Genomic DNA was extracted from a 4 ml aliquot of the centrifuged consortium sample using the soil-specific FastDNA SPIN Kit (MP Biomedicals, France). Using a Nanodrop (ND-1000, USA) spectrophotometer, we obtained concentration measurements of the isolated DNA replicates, yielding values of 214.2 ng/ml with a 260/280 ratio of 1.88 and 203.7 ng/ml with a 260/280 ratio of 1.87. Electrophoresis was used to determine the DNA's purity (Bio-Rad, India). The TRIzol Plus RNA Purification Kit (Lift Technologies, USA) was used right away after sampling for total RNA isolation. At 4 °C, a 12 ml consortium sample that contained about 1500 mg of dry thermophilic consortia sample was centrifuged for 5 min at 13,000 g. Following the manufacturer's directions, independent cell lysis and total RNA precipitation on three biomass pellets were carried out. The Amplification Grade DNase I Kit (Sigma, USA) was used to eliminate the genomic DNA. The integrity of the extracted RNA was evaluated using a Bioanalyzer (Agilent Technologies, USA). Subsequently, the creation of first- and second-strand cDNA was achieved using the Superscript III First-Strand Synthesis SuperMix (Invitrogen, USA) and the Second-Strand cDNA Synthesis Kit (BeyoTime, China).

### 4.4 Library preparation and sequencing

The creation of DNA and cDNA libraries as well as the sequencing process were outsourced to Hyderabad, India's AgriGenome Labs Pvt Ltd. The Bioruptor^Ⓡ^ bath sonicator (Diagenode) was used to shear 20 ng of DNA in a 15–90 s ON–OFF cycle that was performed seven times to build libraries using the NEBNext^Ⓡ^ UltraTM DNA Library Prep Kit for Illumina^Ⓡ^. A BioAnalyzer High Sensitivity Chip was used to evaluate the libraries' size and quality. Sequencing was performed on diluted libraries (2 nM) using Illumina's HiSeq 2500 sequencer, employing 2 × 150 bp paired-end sequencing.

### 4.5 Taxonomic and functional analysis

In the analysis of the fastq files, we assessed several parameters, including the distribution of base quality scores, average base content per read, sequence quality scores, GC distribution in the reads, over-represented sequences, PCR amplification issues, and trimming of adapter. After reviewing the quality report, we conducted sequence trimming to retain exclusively high-quality sequences (QC > 20) for subsequent analysis, while excluding any sequences of low quality (QC ≤ 20). Adapter trimming was accomplished using Cutadapt (v 3.4) [3]. For *de novo* assembly, MetaSPAdes (v 3.15.3) [4] was employed, which utilizes a de Bruijn graph-based approach and incorporates optional modules for various processing and assembly steps. The resulting contigs from the assembly were subjected to open reading frame (ORF) prediction using MetaGeneAnnotator (MGA) [5]. MGA is a gene-finding program specifically designed for the coding regions identification and differentiates them from noncoding DNA. The predicted ORFs were then used for taxonomic classification and functional annotation. For functional annotation, the ORFs were aligned against the non-redundant (NCBI-nr) database (Accessed 12 December 2022) using DIAMOND (v0.7.9.58) [6] to identify their functional characteristics. The alignment file, along with the filtered ORFs, was subsequently utilized as input for functional annotation using MEtaGenome Analyzer (MEGAN6) software [7]. MEGAN6 is a powerful and efficient tool for analyzing metagenomic and metatranscriptomic data at both taxonomic and functional levels. It enables comprehensive analysis and comparison of datasets, facilitating insights into the taxonomic and functional composition of the metagenomic data.

## Ethics Statements

The authors did not use animal or human experimental materials and thus are not subject to ethical concerns.

## CRediT authorship contribution statement

**Sangita Dixit:** Conceptualization, Data curation, Formal analysis, Investigation, Methodology, Visualization, Writing – original draft, Writing – review & editing. **Kalpana Sahoo:** Conceptualization, Formal analysis, Methodology, Writing – review & editing. **Mahendra Gaur:** Conceptualization, Data curation, Formal analysis, Methodology, Visualization, Writing – review & editing. **Enketeswara Subudhi:** Conceptualization, Project administration, Resources, Supervision, Writing – original draft, Writing – review & editing.

## Data Availability

Metatranscriptomics of consortium sample: Deulajhari Hotspring (Original data) (NCBI) Metagenomics of consortium sample: Deulajhari Hotspring (Original data) (NCBI)

## Appendix Supplementary materials

Image, application 1

Image, application 2

Image, application 3
